# Diversity of dengue virus serotypes in Nepal during the 2022 outbreak

**DOI:** 10.1186/s41182-025-00707-7

**Published:** 2025-12-06

**Authors:** Bimalesh Kumar Jha, Priya Jha, Lilee Shrestha, Bal Krishna Awal, Ranjan Raj Bhatt, Runa Jha

**Affiliations:** National Public Health Laboratory Teku, Kathmandu, Nepal

**Keywords:** Dengue, RT PCR, Serotype, Outbreak, Province

## Abstract

**Background:**

In 2022, a widespread dengue outbreak was reported in Nepal, with 4,593 cases reported by August escalating to 53,951 cases and 62 deaths by November. The caseload was significantly higher than in 2019, when the outbreak was last officially recorded with 17,999 cases and 6 fatalities.

**Aims and objectives:**

This study aimed to identify Nepal’s circulating dengue virus serotypes during the 2022 outbreak and analyze their distribution across different regions and hospitals.

**Materials and methods:**

To better understand the circulating dengue virus serotypes, a study was conducted in collaboration with the Seven Provincial Hospital and National Public Health Laboratory (NPHL) with the Coordination of Epidemiology Diseases Control Division (Nepal). This study involved collecting and testing blood samples from various Provincial hospitals across Nepal, covering all the geographical patterns of Nepal.

**Results:**

The results revealed a predominance of serotype 1, followed by serotypes 2 and 3, with some mixed infections. The findings underscore the diverse dengue serotype circulation and the potential for severe dengue infections in Nepal.

**Conclusion:**

The 2022 dengue outbreak in Nepal was primarily driven by DENV-1, followed by DENV-2 and DENV-3. The detection of mixed infections and the absence of DENV-4 warrant further investigation to understand the transmission dynamics and potential for severe dengue cases.

## Introduction

In Nepal and other tropical and subtropical regions, dengue is a major public health concern. Four distinct but closely related dengue virus serotypes (DENV-1, DENV-2, DENV-3, and DENV-4) cause the disease, which is spread by Aedes mosquitoes. As of November 30, 2022, there were 53,951 cases of dengue and 62 deaths in Nepal [1]. The current dengue epidemic in Nepal is the largest to date, as evidenced by the highest cumulative number of cases ever recorded. The first dengue case was discovered in Nepal in 2004 [2]. After thereafter, there were isolated outbreaks in 2010, 2013, 2016, and 2017, which caused the incidence to rise from 686 to 2,111 each year. There was a notable outbreak in 2019, with 17,992 cases reported. Infections with dengue have been documented in recent years in both newer, more populated districts and the nation’s higher mountainous regions, indicating that the infection’s spread has accelerated [3]. There was a notable outbreak in 2019, with 17,992 cases reported. Infections with dengue have been documented in recent years in both newer, more populated districts and the nation’s higher mountainous regions, indicating that the infection’s spread has intensified [4]. In 2024, Nepal experienced a significant dengue outbreak, with over 28,000 reported cases across 76 districts and 12 fatalities. Notably, the virus spread to high-altitude regions, including Solukhumbu district, home to Mount Everest, and the capital city, Kathmandu, which reported over 4,000 cases. Despite the severity of the outbreak, authorities discontinued preventive measures such as mosquito search and destroy campaigns, contributing to the rise in infections. This lack of intervention led to public resignation, with many feeling helpless against the spread of the virus. The surge in dengue cases has been linked to climate change and urbanization, which have expanded the habitats of dengue-carrying mosquitoes to higher elevations previously unaffected by the virus.

The greatest dengue outbreak in Nepali history occurred in 2022 [1, 3]. The Epidemiology and Disease Control Division (EDCD) of the MoHP’s Department of Health Services employed a multisectoral approach to address this issue, including “search and destroy” programs and increasing public awareness. The EDCD organized district and local responses to curb the increasing number of dengue cases and facilitated a virtual meeting involving provincial, district, local, and other stakeholders [1, 4]. All seven provinces of the country, 60 districts, and 139 affected municipalities received funding for the “search and destroy” operations from the EDCD. In response to the increasing dengue incidence, the district health offices in Lalitpur and Kathmandu intensified mass dengue search and destroy operations and awareness-raising campaigns in the last week of August, collaborating closely with the metropolitan areas ^(1, 4)^. Later, the entire country was included in the campaign. However, due to insufficient vector surveillance, molecular analysis of circulating DENV serotypes, and an inefficient early-warning system, it was challenging to accurately quantify the viral spread across regions and altitudes. Consequently, the control measures were not scheduled and executed in a timely manner. Clinical manifestations of dengue include severe dengue without warning signs, dengue with warning signs (e.g., abdominal pain, persistent vomiting, clinical fluid accumulation, mucosal bleeding, lethargy, liver enlargement, and increasing hematocrit with decreasing platelets) and severe dengue (includes severe plasma leakage, severe bleeding, or severe organ involvement such as liver, heart, or CNS dysfunction).

A major dengue outbreak was recorded in Nepal in 2022, which surpassed the 2019 outbreak in terms of the number of cases and deaths. Understanding the circulating dengue virus serotypes is essential to improve public health responses and clinical management, as different serotypes have varying effects on disease severity and transmission dynamics [5].

The purpose of this study was to determine which dengue virus serotypes were prevalent in Nepal during the 2022 outbreak and to examine how they were distributed among various hospitals and geographical areas.

## Methods and methodology

### Study sites, sample collection, and ethical declarations

Samples from 380 patients who visited nine hospitals spread throughout all seven provinces of Nepal between September and October 2022 the busiest months of the outbreak that year were used in this cross-sectional descriptive and retrospective analysis. A list of the participating websites was provided. The Nepal Health Research Council gave its approval to the study protocol (Reg. no. 405/2024). All adult patients or the parent or legal guardian of a child under the age of 18 years provided written informed consent.

### Ethics statement

The Nepal Health Research Council Ethical Review Board gave its approval to this study (reg. 405/2024). Based on the fundamental ideas of the Nuremberg Code, the World Medical Association Declaration of Helsinki, the Council of International Organization of Medical Sciences, the World Health Organization (WHO), the International Conference on Harmonization and Guidelines for Good Clinical Practice, and the International Ethical Guidelines for Biomedical Research Involving Human Subjects, the Nepal Health Research Council currently follows this version of the guidelines. We are not personally associated with any of the patients because this is a retrospective study and all of the data evaluated came from the Vaccine-Preventable Diseases Laboratory throughout the study period. This study is a laboratory-based retrospective study conducted at National Public Health Laboratory. Ethical approval was obtained from Ethical Review Committee, Nepal Health Research Council, and no individual consent from participants was taken as data were extracted from Vaccine-Preventable Diseases Department with the approval of Director of the National Public Health Laboratory. All the samples were anonymized and only code number generated in the laboratory were used for analysis. Therefore, written informed consent is deemed unnecessary for this research as this study was approved by both NHRC and NPHL.

### Study population, enrollment criteria, and sample collection

Samples from 380 patients who visited nine hospitals spread throughout all seven provinces of Nepal between “September and October 2022” the busiest months of the outbreak that year were used in this cross-sectional descriptive and retrospective analysis. Both inpatients and outpatients from various hospitals and sentinel locations across all seven provinces were among the enrolled patients. Two or more of the following symptoms had to be present in order to be eligible: nausea, body/muscle or stomach discomfort, vomiting, rashes, or a temperature more than 38 °C at the time of sample collection. The attending physician documented clinical and demographic information, and the geospatial distribution of dengue patients was represented using ArcMap 10.4 software (ArcGIS). At registration, single blood samples (3–5 mL) were taken, and the sera were centrifuged right away and kept at −20 °C.

### Sample collection

Blood samples were collected from nine hospitals across various provinces of Nepal, as listed in Table 1. The sample collection was conducted between 23rd September 2022 and 21st October 2022.

A total of 380 blood samples were collected, with specific breakdowns provided in Table 1.

A retrospective, descriptive, and cross-sectional study was carried out at the Vaccine-Preventable Diseases Laboratory (VPD), National Public Health Laboratory (NPHL), Kathmandu, Nepal for the period of the high peak of cases. The data were obtained from the Vaccine-Preventable Diseases Laboratory for the research purpose on 1st March 2023. Serum samples totaling 380 were obtained from NPHL and the selected Provincial Hospital. Within 24 h after being collected at the sites, the samples were brought to NPHL in a cold chain box and stored at 4 °C until processing. All the samples tested positive for the NS1 antigen using immunochromatography. The Standard Q Dengue Duo test from SD BIOSENSOR has a sensitivity of 92.9% and a specificity of 98.7% for the NS1 antigen.

### NS1 antigen detection and anti-IgM/IgG DENV serology

According to the manufacturer’s instructions, serum samples were examined for the presence of DENV NS1 antigen and anti-DENV IgM and IgG using Rapid Diagnostic Test kits that were accessible at the hospital and designated sentinel site. Inconclusive samples were retested; if the results were still unclear, the sample was deemed negative. The National Public Health Laboratory Teku received the samples.

### Sample processing and serotype detection

Each sample was aliquoted into a microcentrifuge tube from its in-plane tube. One was used for RNA extraction, and the other was maintained at −70 °C. The Unimedica Automated Nucleic Acid Extraction System was used in accordance with the manufacturer’s instructions to extract nucleic acids from dengue virus-positive samples. To guarantee full lysis, 200 µL of serum, 200 µL of lysis buffer, and 20 µL of proteinase K were first combined in a biosafety cabinet and allowed to sit at room temperature for 10 min. After that, the mixture, the magnetic bead cartridge, and the required reagents—binding buffer, wash buffers, and elution buffer—were loaded into the Unimedica system. Using a master mix made with primers, probes, and superscript III enzymes for tests (n = 380) and controls, reverse transcriptase real-time PCR was carried out (BIORAD-CFX96 TouchTM Real-Time PCR Detection System) in accordance with the Centers for Disease Control and Prevention’s (CDC) USA guideline for DENV serotype identification. If the amplification of the probes—the FAM (blue), VIC (green), Texas Red (red), and Cy5 (purple) curves—were amplified within CT value 37, the results were considered as positive for DENV-1, 2, 3, and 4.

All procedures used in this study were approved by the Nepal Health Research Council and carried out in accordance with established rules and regulations. The research methods complied with the Helsinki Declaration’s suggested protocols and ethical guidelines.

## Results

### Sample testing and serotype distribution

All 380 samples collected from the sentinel hospitals were tested for dengue virus serotypes. The distribution of samples and their testing status are summarized in Table 2.

### Serotype analysis

**Fig. 1:**
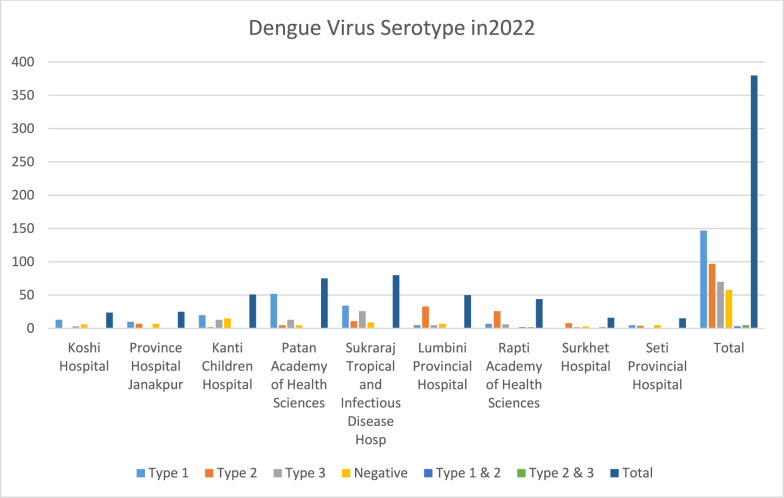
X-Axis dengue virus serotype in 2022 of different provinces and Y-Axis total number of samples tested

Table 3 displays the findings of the serotyping analysis. DENV-1 was the most common serotype, with DENV-2 and DENV-3 following closely behind. There were also reports of mixed infections, with some samples exhibiting the presence of multiple serotypes. Interestingly, 58 samples had negative dengue virus tests, suggesting that other viral infections or false negatives could have occurred in Fig. 1.

## Discussion

The results indicate that DENV-1 was the most prevalent serotype during the 2022 dengue outbreak in Nepal. The presence of multiple serotypes, including mixed infections, suggests a complex epidemiological landscape and the potential for severe disease manifestations. The absence of DENV-4 in this study contrasts with previous reports of its presence in Nepal, highlighting the dynamic nature of dengue virus circulation.

Dengue cases are rising worldwide, particularly in endemic countries in South America and Southeast Asia, such as Nepal, where cases in the 2022 outbreak were three times higher than in 2019. This study offers important insights into the distribution and prevalence of dengue virus serotypes in Nepal, which can guide public health strategies, including vector control measures and targeted interventions to lessen the impact of future dengue outbreaks [6]. Europe is among the new regions where DENV infection is expanding. In the United States, cases of locally acquired dengue have been documented in Texas, Arizona, Hawaii, and California, and during the 2022–2023 dengue season, over 1000 DENV infections (71 locally acquired) were reported in Florida [7]. Programs for scalable real-time DENV genome surveillance will be necessary to address this public health concern. This research is a component of a continuous endeavor to develop such a program. Dengue has expanded somewhat around the world from tropical and subtropical areas to temperate ones. In 2016, 3% of all cases reported in Nepal came from the temperate zone, and in 2022, 50% did [8]. Participants in our study came from five of Nepal’s seven provinces, and the majority lived in higher elevation temperate districts. This is in line with a 2023 report from Nepal’s Ministry of Health and Population that revealed dengue cases in all seven provinces and 77 districts for the first time, with the highest number of cases occurring in higher-elevation temperate districts [9]. This spread to temperate regions at higher elevations is probably caused by a confluence of climate change, mosquito adaptation, and DENV development [10]. Our results suggest regional transmission dynamics between China, the world’s most populous country; India, the second most populous country and one with a high dengue burden; and Nepal. China and India share a border with Nepal to the north and south, respectively. However, the Himalayan range separates China and Nepal, suggesting that DENV transmission into Nepal is more likely to come from India [11]. However, more Chinese tourists are traveling to big cities, which could help spread DENV from the north. Although severe dengue is typically seen in secondary DENV infections, it was recently reported in India during primary infections, indicating that DENV-host interactions may vary depending on the population, geographic location, and other viral or environmental factors [12]. A cohort of 619 children with confirmed dengue, ages 2 months to 16 years, who were seen at three clinical sites between 2012 and 2018, was included in the Indian study [13]. The WHO 2009 recommendations for DENV infection were used to assess the severity of the disease, and the ratio of DENV-specific IgM to IgG as determined by ELISA was used to categorize individuals as having primary or secondary infections [14]. A 2022 study from Dhaka found that the WHO 2009 classification was useful in identifying severe cases but had limitations in predicting fatal outcomes Similar to Nepal, DENV-2 was the most dominant serotype, leading to a high number of severe cases. Some cases categorized as “Dengue with warning signs” progressed rapidly to severe dengue, suggesting a need for earlier intervention criteria [15]. The study contradicts the widely held belief that severe dengue is mostly linked to secondary infections by showing that disease severity during both primary and secondary infection was unaffected by the infecting DENV serotype, neutralizing titers, or clinical location. A 2021 study in Tamil Nadu suggested that the WHO 2009 system was effective, but some patients with severe organ involvement (hepatic and renal dysfunction) were missed in early classifications DENV-2 and DENV-3 were co-circulating, with reports of severe dengue and dengue shock syndrome [16]. Therefore, it is evident that both local and more extensive regional surveillance systems are required. Our multi-year global partnership shows that it is possible to establish mutually advantageous on-site genetic surveillance systems in low-income nations. A 2023 study from Colombo reported that shock syndrome and plasma leakage were the most critical determinants of mortality Predominantly DENV-3, but DENV-2 was also detected in certain regions, contributing to hospitalizations [17]. The influence of the emerging research capability in Nepal will be extensive. Molecular surveillance identified DENV-2 as the most dominant serotype in Nepal in 2019 [18]. It advances the careers of academics in both high- and low-income nations while offering a singular chance to investigate the evolution and spread of DENV in South and East Asia.

## Conclusion

DENV-1 was the main cause of the dengue outbreak in Nepal in 2022, with DENV-2 and DENV-3 following closely behind. To comprehend the dynamics of transmission and the possibility of severe dengue cases, more research is necessary in light of the finding of mixed infections and the lack of DENV-4. For dengue control and prevention initiatives in Nepal to be successful, ongoing surveillance and serotyping are crucial.

## Data Availability

Vaccine-Preventable Diseases Laboratory datasets analyzed during the current investigation are accessible; the remaining datasets will be supplied by corresponding authors upon reasonable request. All relevant data are included in the manuscript.

## Appendix 1. Standard operating procedure for blood sample collection for dengue virus serotyping

See Tables 1, 2 and 3 and Fig. 1.

**Table 1:** Breakdown of collected samples

S. N	Hospital	Total samples collected	Period of sample collection
1	Koshi Hospital	24	2079/06/07 to 2079/06/11
		18	2079/06/11 to 2079/06/26
2	Janakpur Hospital	25	2079/06/11 to 2079/06/28
3	Patan Academy of Health Sciences	75	2079/06/10 to 2079/06/11
4	Sukraraj Tropical and Infectious Disease Hosp	80	2079/06/11 to 2079/06/11
5	Kanti Children Hospital	51	2079/06/13 to 2079/06/28
6	Lumbini Provincial Hospital	50	2079/06/14 to 2079/06/27
7	Rapti Academy of Health Sciences	25	2079/06/07 to 2079/06/10
		19	2079/06/11 to 2079/06/22
8	Surkhet Hospital	16	2079/06/31 to 2079/07/04
9	Seti Provincial Hospital	15	2079/06/09 to 2079/06/25
		6	2079/06/09 to 2079/06/10
Total	380

**Table 2:** Samples received and tested

Province	Hospital	Samples received	Samples tested
Province 1	Koshi Hospital	24	24
Madhesh Province	Province Hospital Janakpur	25	25
Bagmati Province	Kanti Children Hospital	51	51
	Patan Academy of Health Sciences	75	75
	Sukraraj Tropical and Infectious Disease Hosp	80	80
Gandaki Province	Pokhara Academy of Health Sciences	0	0
Lumbini Province	Lumbini Provincial Hospital	50	50
	Rapti Academy of Health Sciences	44	44
Karnali Province	Surkhet Hospital	16	16
Sudurpaschim Province	Seti Provincial Hospital	15	15
Total	**-**	**380**	**380**

**Table 3:** Dengue virus serotypes identified by hospital

Site	Dengue type 1	Dengue type 2	Dengue type 3	Dengue negative	Dengue type 1 and 2	Dengue type 2 and 3	Total
Koshi Hospital	13	1	3	6	1	0	24
Province Hospital Janakpur	10	7	1	7	0	0	25
Kanti Children Hospital	20	2	13	15	0	1	51
Patan Academy of Health Sciences	52	5	13	5	0	0	75
Sukraraj Tropical and Infectious Disease Hosp	34	11	26	9	0	0	80
Lumbini Provincial Hospital	5	33	5	7	0	0	50
Rapti Academy of Health Sciences	7	26	6	1	2	2	44
Surkhet Hospital	1	8	2	3	0	2	16
Seti Provincial Hospital	5	4	1	5	0	0	15
Total	147	97	70	58	3	5	380

## Declarations

### Limitation of study

The study is limited to serotypic classification only, no sequencing and cytogenetic study were performed due to unavailability of reagents. In addition, for privacy, no demographic information of patients was revealed.

## References

[1] Rijal KR, Adhikari B, Ghimire B, Dhungel B, Pyakurel UR, Shah P, et al. Epidemiology of dengue virus infections in Nepal, 2006–2019. Infect Dis Poverty. 2021;10:1–10. 33858508 10.1186/s40249-021-00837-0 PMC8047528

[2] PANT, S. K. (2017). *SEROLOGICAL STUDY OF DENGUE VIRUS INFECTION IN DANG DISTRICT OF NEPAL* (Doctoral dissertation, Tribhuvan University).

[3] Pokharel P, Khanal S, Ghimire S, Pokhrel KM, Shrestha AB. Frequent outbreaks of dengue in Nepal–causes and solutions: a narrative review. IJS Global Health. 2023;6(5):e0351.

[4] Poudel P, Khatri R, Bhatt L, Thapa P, Mishra RK, Tuladhar S, Panahi E. Baseline Status of Basic Health Service Delivery, 2022 Nepal DHS and 2021 Nepal HFS. DHS Further Analysis Reports. 2024;157:1.

[5] Gyawali N, Johnson BJ, Dixit SM, Devine GJ. Patterns of dengue in Nepal from 2010–2019 in relation to elevation and climate. Trans R Soc Trop Med Hyg. 2021;115(7):741–9. 33197254 10.1093/trstmh/traa131

[6] Sah R, Siddiq A, Padhi BK, Mohanty A, Rabaan AA, Chandran D, Dhama K. Dengue virus and its recent outbreaks: current scenario and counteracting strategies. Int J Surg. 2023;109(9):2841–5. 36906765 10.1097/JS9.0000000000000045 PMC10498890

[7] Napit R, Elong Ngono A, Mihindukulasuriya KA, Pradhan A, Khadka B, Shrestha S, Manandhar KD. Dengue virus surveillance in Nepal yields the first on-site whole genome sequences of isolates from the 2022 outbreak. BMC Genom. 2024;25(1):998. 10.1186/s12864-024-10879-x PMC11515306 39449117

[8] Pandey DP, Thapa NB. Analysis of news media-reported snakebite envenoming in Nepal during 2010–2022. PLoS Negl Trop Dis. 2023;17(8):e0011572. 37639403 10.1371/journal.pntd.0011572 PMC10491300

[9] Kramer IM, Pfenninger M, Feldmeyer B, Dhimal M, Gautam I, Shreshta P, Waldvogel AM. Genomic profiling of climate adaptation in Aedes aegypti along an altitudinal gradient in Nepal indicates nongradual expansion of the disease vector. Mol Ecol. 2023;32(2):350–68. 36305220 10.1111/mec.16752

[10] Dhimal M, Gautam I, Joshi HD, O’Hara RB, Ahrens B, Kuch U. Risk factors for the presence of chikungunya and dengue vectors (Aedes aegypti and Aedes albopictus), their altitudinal distribution and climatic determinants of their abundance in central Nepal. PLoS Negl Trop Dis. 2015;9(3):e0003545. 25774518 10.1371/journal.pntd.0003545 PMC4361564

[11] Lahondère C, Bonizzoni M. Thermal biology of invasive Aedes mosquitoes in the context of climate change. Curr Opin Insect Sci. 2022;51:100920. 35421621 10.1016/j.cois.2022.100920

[12] Sirisena PDNN, Mahilkar S, Sharma C, Jain J, Sunil S. Concurrent dengue infections: epidemiology & clinical implications. Indian J Med Res. 2021;154(5):669–79. 35532585 10.4103/ijmr.IJMR_1219_18 PMC9210535

[13] Aggarwal C, Ahmed H, Sharma P, Reddy ES, Nayak K, Singla M, Murali-Krishna K. Severe disease during both primary and secondary dengue virus infections in pediatric populations. Nat Med. 2024;30(3):670–4. 38321219 10.1038/s41591-024-02798-x PMC7617637

[14] Puschnik A, Lau L, Cromwell EA, Balmaseda A, Zompi S, Harris E. Correlation between dengue-specific neutralizing antibodies and serum avidity in primary and secondary dengue virus 3 natural infections in humans. PLoS Negl Trop Dis. 2013;7(6):e2274. 23785536 10.1371/journal.pntd.0002274 PMC3681624

[15] Htun TP, Xiong Z, Pang J. Clinical signs and symptoms associated with WHO severe dengue classification: a systematic review and meta-analysis. Emerg Microb Infect. 2021;10(1):1116–28. 10.1080/22221751.2021.1935327 PMC8205005 34036893

[16] Sirisena PDNN, Mahilkar S, Sharma C, Jain J, Sunil S. Concurrent dengue infections: epidemiology & clinical implications. Indian J Med Res. 2021;154(5):669–79. 35532585 10.4103/ijmr.IJMR_1219_18 PMC9210535

[17] Fernando L, Wickramasinghe VP, Kalra P, Kastner R, Gallagher E. Epidemiological burden of dengue in Sri Lanka: a systematic review of literature from 2000–2020. IJID Regions. 2024;1:100436. 10.1016/j.ijregi.2024.100436 PMC11462220 39386112

[18] Katuwal N, Shrestha A, Ranjitkar U, Jakibanjar S, Madhup SK, Tamrakar D, Shrestha R. Molecular investigation of denv serotypes in the dengue outbreak of 2022 in nepal. Medrxiv. 2023;2023:1–05. 39324467

[19] WHO, 2022. Dengue and severe dengue. https://www.who.int/news-room/fact-sheets/detail/dengue-and-severe-dengue.

[20] EDCD, 2019. Dengue Virus Serotype Circulation in Nepal. Epidemiology and Disease Control Division, Ministry of Health and Population, Nepal. EDCD, 2019. Dengue Virus Serotype Circulation in Nepal.

